# Diagnóstico ecográfico de nefrocalcinosis en un lactante con vómitos recurrentes

**DOI:** 10.1016/j.aprim.2026.103458

**Published:** 2026-02-13

**Authors:** Marta Cecilia Carrasco Hidalgo-Barquero, Luis Ortiz-Peces, Luis Ortiz-González

**Affiliations:** a Unidad de Nefrología Pediátrica, Hospital Materno-Infantil de Badajoz, Badajoz, España; b Servicio de Cirugía Oral y Maxilofacial, Hospital Universitario La Paz, Madrid, España; c Departamento de Ciencias Biomédicas, Facultad de Medicina y Ciencias de la Salud, Universidad de Extremadura, Badajoz, España

La nefrocalcinosis se caracteriza por la presencia de depósitos de oxalato cálcico y fosfato cálcico en el parénquima renal, habitualmente en las pirámides medulares. Puede tener un origen multifactorial, que incluye causas genéticas (tubulopatías), endocrinas (hipercalcemia), farmacológicas, infecciosas e inflamatorias. En lactantes, las causas de nefrocalcinosis son similares a las descritas en otras edades, aunque predominan las tubulopatías hereditarias —como la acidosis tubular renal distal, el síndrome de Bartter y la hipomagnesemia familiar con hipercalciuria y nefrocalcinosis (HFHN)—, junto con la exposición a diuréticos del asa en prematuros y la hipervitaminosis D por suplementación excesiva. También pueden observarse casos asociados a hiperoxaluria primaria, riñón esponjoso medular o infecciones urinarias recurrentes, por lo que resulta esencial realizar un estudio metabólico y genético completo ante hallazgos ecográficos bilaterales de nefrocalcinosis para identificar la causa subyacente y orientar el tratamiento^1^, ^2^.

Entre las causas genéticas más relevantes se encuentra la HFHN, una tubulopatía hereditaria del asa de Henle causada por mutaciones en los genes *CLDN16* o *CLDN19*, que codifican las proteínas claudina-16 y claudina-19, implicadas en el transporte paracelular de magnesio y calcio. Estas proteínas son esenciales para mantener el equilibrio iónico tubular. La enfermedad, que presenta un patrón de herencia autosómica recesiva, se manifiesta habitualmente en la infancia temprana y, sin tratamiento adecuado, puede progresar a insuficiencia renal crónica y requerir tratamiento sustitutivo renal en la segunda o tercera décadas de la vida^3^.

La semiología clínica es muy variada, desde formas asintomáticas hasta manifestaciones inespecíficas como vómitos persistentes, irritabilidad o fallo de medro, lo que dificulta su diagnóstico precoz. En muchos casos, los hallazgos ecográficos constituyen el primer signo de la enfermedad^4^.

Con el diagnóstico temprano de nefrocalcinosis mediante ecografía, seguido de estudios analíticos y genéticos que permiten confirmarla, se pueden iniciar las medidas necesarias para mejorar y enlentecer la progresión del daño renal^5^. No existe un tratamiento específico, por lo que el manejo se centra en medidas de soporte: suplementación con magnesio (sin pretender normalizar los valores), citrato potásico (para reducir o enlentecer la nefrocalcinosis, aunque con evidencia limitada), metabolitos de vitamina D y, como pilar fundamental, mantener una adecuada hidratación. Estas intervenciones buscan disminuir la pérdida tubular de magnesio y calcio, reducir la formación de depósitos cálcicos y preservar la función renal a largo plazo^5^, ^6^.

Presentamos el caso de un lactante de 10 meses que presentaba vómitos recurrentes de 1,5 meses de evolución y fallo de medro, al que realizamos una ecografía abdominal, donde se objetivó una marcada hiperecogenicidad de las pirámides renales, bilateral y simétrica, hallazgos compatibles con nefrocalcinosis medular (Figura 1, Figura 2, y vídeo). Se remitió al hospital, donde se objetivaron datos de daño renal (creatinina sérica elevada para su edad e hipomagnesemia), por lo que se sospechó la presencia de una HFHN. Se realizó estudio genético y se identificó la presencia de una variante patogénica en el gen *CLDN19*, que confirmó el diagnóstico clínico hospitalario de sospecha. Se inició tratamiento con suplementos de magnesio, citrato potásico e hidratación oral abundante. Actualmente, el paciente se mantiene en seguimiento con función renal estable.

**Figura 1:**
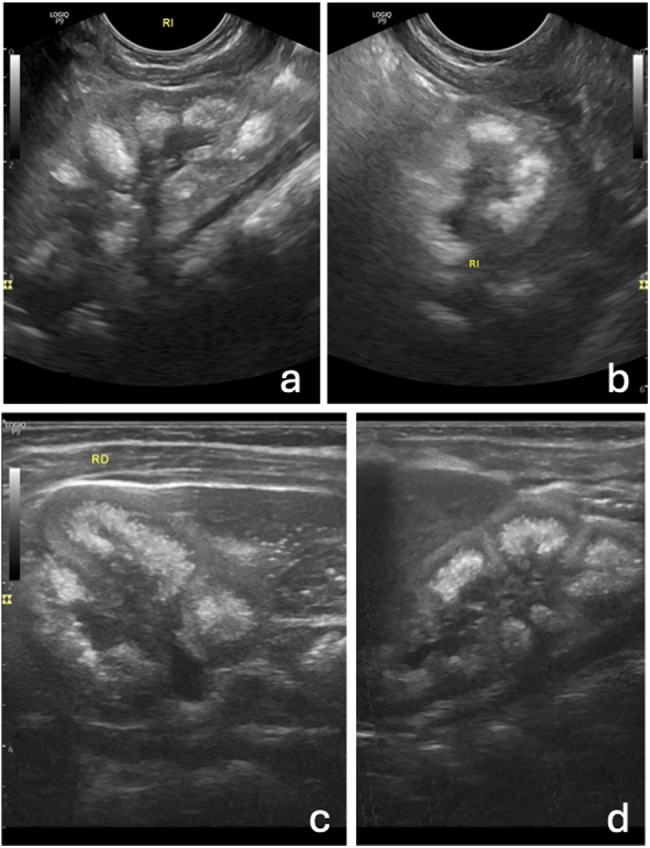
Imágenes ecográficas de riñones con nefrocalcinosis. a y b) Cortes longitudinal-oblicuo (a) y transversal (b) del abdomen a nivel de hipocondrio izquierdo, con sonda microconvex, donde se objetiva el riñón izquierdo (RI) con hiperecogenicidad medular piramidal, sugerente de nefrocalcinosis. c y d) Cortes transversal (c) y longitudinal-oblicuo (d) del abdomen a nivel de la pared posterior derecha, con sonda lineal de alta frecuencia (8-12 MHz), donde se observa el riñón derecho (RD) con hallazgos similares de hiperecogenicidad medular.

**Figura 2:**
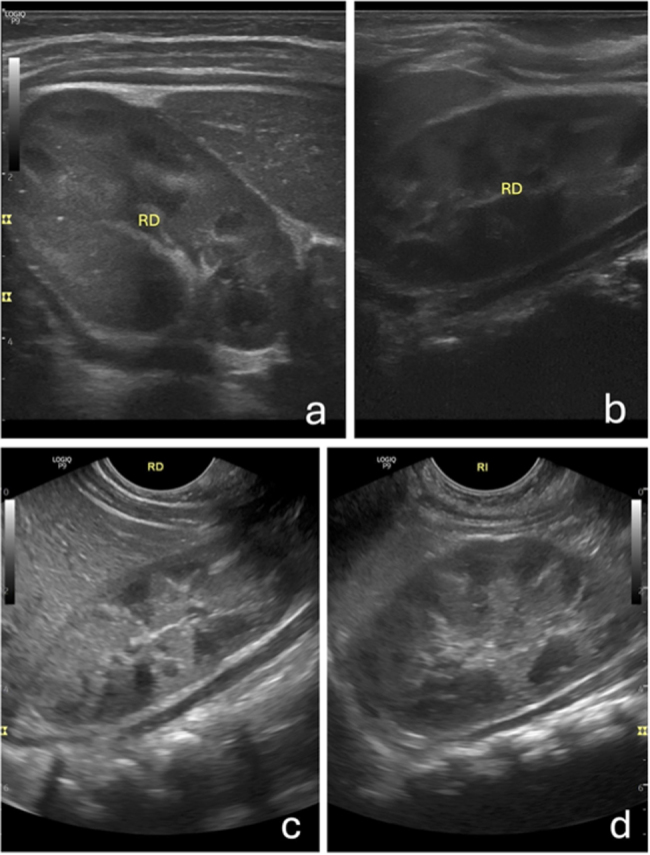
Imágenes ecográficas de riñones normales (compárese con las imágenes de nefrocalcinosis de la figura 1). a y b) Cortes transversal (a) y longitudinal-oblicuo (b) del abdomen a nivel de la pared posterior, con sonda lineal de alta frecuencia (8-12 MHz), donde se objetiva el riñón derecho (RD) normal, con pirámides hipoecogénicas. c y d) Cortes longitudinal-oblicuos del abdomen a nivel de los hipocondrios derecho (c) e izquierdo (d), con sonda microconvex, donde se observan los riñones derecho (RD) e izquierdo (RI), respectivamente, con hipoecogenicidad piramidal.

Destacamos el papel de la ecografía clínica como herramienta de apoyo para el diagnóstico precoz de nefrocalcinosis en lactantes con síntomas inespecíficos. Su uso aporta gran valor y amplía la capacidad resolutiva del médico en Atención Primaria. En este caso, el estudio ecográfico abdominal permitió orientar el diagnóstico hacia una tubulopatía hereditaria y gestionar una derivación hospitalaria más eficiente. La integración sistemática de esta técnica en la práctica clínica puede mejorar de manera significativa la calidad asistencial y el pronóstico a largo plazo de muchos pacientes^6^.

## Financiación

Los autores manifiestan que no han recibido financiación alguna para la elaboración del manuscrito.

## Consideraciones éticas

Los autores confirman que se han obtenido todos los consentimientos requeridos por la legislación vigente para la publicación de cualquier dato personal o imágenes de pacientes, sujetos de investigación u otras personas que aparecen en los materiales enviados a Elsevier, se han realizado todos los procedimientos éticos y se han respetado los derechos de privacidad de los sujetos humanos.

## Conflicto de intereses

Ninguno.

## Appendix B Material adicional

**Vídeo**. Imágenes dinámicas de ambos riñones con nefrocalcinosis (RD y RI) obtenidas mediante cortes longitudinales-oblicuos, con sonda microconvex, a nivel de ambos hipocrondrios.

## References

[1] Zheng J., Cao J., Chen L., Xia X. (2025). Clinical characteristics of nephrocalcinosis in a tertiary children's hospital. Front Pediatr..

[2] Ghanem H., Hassan M., Swideah S., Wannous H. (2025). First retrospective study on pediatric nephrocalcinosis in Syria: clinical symptoms and causes. Front Pediatr..

[3] Vall-Palomar M., Madariaga L., Ariceta G. (2021). Familial hypomagnesemia with hypercalciuria and nephrocalcinosis. Pediatr Nephrol..

[4] Woo H.A., Lee H., Choi Y.H., Min J., Kang H.G., Ahn Y.H. (2023). Clinical outcomes of nephrocalcinosis in preschool-age children: association between nephrocalcinosis improvement and long-term kidney function. Front Pediatr..

[5] García-Nieto V.M., Claverie-Martín F., Perdomo-Ramírez A., Cárdoba-Lanus E., Ramos-Trujillo E., Mura-Escorche G. (2020). Considerations about the molecular basis of some kidney tubule disorders in relation to inbreeding and population displacement. Nefrologia (Engl Ed)..

[6] Juárez Castillo A., Ruiz Moreno M., González Peregrina J., Belando Peñalver Á. (2024). Uso de la ecografía clínica en Atención Primaria. Estudio prospectivo multicéntrico [Use of clinical ultrasound in primary care: Multicenter prospective study]. Aten Primaria..

